# *In Vitro* Antibacterial, Antifungal, Antibiofilm, Antioxidant, and Anticancer Properties of Isosteviol Isolated from Endangered Medicinal Plant *Pittosporum tetraspermum*

**DOI:** 10.1155/2015/164261

**Published:** 2015-05-26

**Authors:** Naif Abdullah Al-Dhabi, Mariadhas Valan Arasu, Thankappan Sarasam Rejiniemon

**Affiliations:** ^1^Department of Botany and Microbiology, Addiriyah Chair for Environmental Studies, College of Science, King Saud University, P.O. Box 2455, Riyadh 11451, Saudi Arabia; ^2^Department of Botany and Biotechnology, AJ College of Science and Technology, Thonnakkal, Trivandrum, Kerala 695 317, India

## Abstract

This study aimed to investigate the *in vitro* antibacterial, antifungal, antibiofilm, antioxidant, and anticancer properties of isosteviol isolated from endangered medicinal plant *Pittosporum tetraspermum*. Pure compound was obtained and characterized by column chromatography followed by ^1^H NMR, ^13^C NMR, IR, and mass spectral analysis. The antimicrobial activities of the compound were assessed by the broth microdilution method and the antioxidant properties were determined using reducing ability assay, DPPH scavenging assay, hydroxyl radical scavenging activity, and superoxide radical scavenging assay. Anticancer study was evaluated by following MTT assay. Column purification and spectrocopical analysis lead to identifying isosteviol from the crude ethyl acetate extract. The compound exhibited significant activity against bacteria such as *Staphylococcus epidermidis* (125 *µ*g/mL), *Staphylococcus aureus* (125 *µ*g/mL), and *Klebsiella pneumoniae* (62.5 *µ*g/mL). The MIC of the compound against *Candida albicans*, *Aspergillus niger*, and *Trichophyton mentagrophytes* was 62.5, 125, and 500 *µ*g/mL, respectively. The compound showed comparatively better antibiofilm activity against *E. coli*, *S. typhi*, and *P. aeruginosa*. Furthermore, it exhibited good antioxidant properties. Anticancer properties of the compound against Vero and MCF7 cell lines were its advantage. Novel isosteviol would be useful to reduce the infectious diseases caused by pathogenic microorganisms or slow the progress of various oxidative stress-related diseases.

## 1. Introduction

Many human died due to the infectious diseases caused by bacteria, fungi, virus, or parasites [1]. An impressive number of modern drugs have been isolated from natural sources, especially, plants that have been used as a source of medicinal agents and produce a diverse range of bioactive metabolites, which are the building blocks for the synthesis of therapeutic drugs, pharmaceuticals, and nutraceuticals [2]. Plants have been used for years in daily life to treat diseases worldwide as well as in the developing countries because of their viable option that could be useful in reducing the side effects associated with conventional antibiotic treatment. Plant metabolites are mainly used in the development of new drugs, especially antimicrobials, which can have therapeutic potential to treat infectious diseases caused by bacteria and fungi [3]. The novel metabolites recovered from medicinal plants were used as anticancer, antidiabetic, antioxidant, anticoagulant, antihypertention, and other cardiovascular diseases. Infectious diseases in human and animal have been in recent years in tropical and subtropical developing countries due to the emergence of pharmaceutical drugs and the development of multiple drug resistance to some of the synthetic drugs. Bacterial resistance to different antibiotics such as *β*-lactams, aminoglycosides, and quinolones is varied in its interacting steps based on the invention of novel compounds belonging to various antibiotic classes, their clinical use, and the characterization of emerging resistance mechanisms. Among the various mechanisms involved in bacterial resistance, the balance of cell wall and cell membrane permeability controls the transport of various molecules, which plays a key role in the influx and efflux of antibiotics and therapeutic compounds, thereby limiting their intracellular concentrations [4]. The NorA efflux plays a significant role in the resistance mechanism against various antibiotics by pumping them out of the cells [5]. The inhibition of an efflux pump can potentially improve by the use of novel antibiotic and thereby decreases the selection of resistance; pharmaceutical companies and research institutes are therefore focusing on identifying novel efflux pump inhibitors (EPIs), which may be clinically useful [6]. At present, there are no EPIs on the market. Therefore it is important to target the NorA efflux pump, which contributes to the emergence of high-level resistance in* Staphylococcus aureus* which causes common nosocomial infections [7].

*Pittosporum tetraspermum* is an endangered medicinal plant mainly distributed in the peninsular parts of the Western Ghats region in India. Three hundred species of* Pittosporum* (Pittosporaceae) are widely distributed worldwide, whereas eleven are observed in India. In* Malayalam* it is widely known as kachappatta, kachapatta, analivegam, and analivetham, and in* Tamil* it is called suneri [8]. The leaves are simple, alternate, spiral, usually crowded at apex, canaliculate, and minutely pubscent when young; the lamina size ranged from 5–10 × 2–4 cm with obovate to oblanceolate shape or narrow elliptic one in nature [9]. This plant is used for the treatment of chronic bronchitis, rheumatism, skin diseases, sprains, leprosy, bruises, sciatica, chest infections, ophthalmia, cutaneous diseases, secondary syphilis, and chronic rheumatism [9]. The bark part is used as narcotic, antidote to snake poison, and also a stimulant. The aim of this study was to test the compound isosteviol isolated from plant* P. tetraspermum*.

## 2. Materials and Methods

### 2.1. Plant Materials and Extraction

The leaves of* P. tetraspermum* were collected from the forest area of the Western Ghats region of Kanyakumari, located at 8.08°N 77.57°E. in February 2012. The plant was identified and authenticated with the accession number DBJ-250 in the Department of Botany and Biotechnology, AJ College of Science and Technology, Thonnakkal, Trivandrum. The leaves were washed thoroughly with distilled water, shade dried, and powdered for the extraction of compounds. Three kilograms of the powder was mixed with hexane, ethyl acetate and chloroform (1 : 3), and vortexes for 24 h for the complete extraction of the compounds. The filtrates were concentrated under reduced pressure using a vacuum rotary evaporator at 40°C and the extract was stored in a refrigerator for further experiments.

### 2.2. Screening and Purification of Active Fraction by Column Chromatography

The crude ethyl acetate extract was screened for antimicrobial activity against Gram positive and Gram negative bacteria by disc diffusion method [10]. The active ethyl acetate extract was chromatographed over silica gel (Acme's 100–200 mesh). The column was eluted with solvents of increasing polarity in order hexane, ethyl acetate, and hexane and their mixtures. All the collected fractions were spotted on a TLC plate over silica gel and eluted to find the single compound with similar retention time (*R*_*f*_). Eight fractions that exhibited similar *R*_*f*_ were pooled together. All the fractions were tested against bacteria and fungi. Fraction 3 exhibited significant antimicrobial activity against tested microbes. The purity of the fraction 3 was checked by HPLC. The compound was identified by an API 4000 Q TRAP tandem mass spectrometer (Applied Biosystems, Foster City, CA) and an electrospray ionisation tandem mass spectrometry (ESI-MS/MS). The MS operating conditions were as follows: ion spray voltage, 5.5 kV; curtain gas (20 psi), nebulizing gas (50 psi) and heating gas (50 psi), and high purity nitrogen (N_2_); heating gas temperature, 550°C; declustering potential (100 V); entrance potential (10 V); spectra scanning range,* m/z* 100–800 (scan time 4.8 s). Functional group of the compounds was determined using Infrared Spectroscopy. Infrared Spectroscopy (IR) was measured using Shimadzu by KBr pellet method. Briefly, carbon tetrachloride was used as a solvent. The solvent was transferred into the KBr crystals and powdered with KBr or Nujomull and analyzed by KBr pellet method. For the NMR analysis, tetra methyl silane was used for the chemical shift to zero on the reading scale. AL-300 MHz, JEOL spectrometer, was used for the NMR spectrum. ^1^H NMR was run at either 300 or 400 MHz and ^13^C NMR at 75 MHz using the solvent signal as reference.

### 2.3. Antimicrobial Activity

#### 2.3.1. Microorganisms

Bacteria including* Bacillus subtilis* (MTCC 441),* Enterococcus faecalis* (ATCC 29212),* Staphylococcus aureus* (ATCC 25923),* Staphylococcus epidermidis* (MTCC 3615),* Escherichia coli* (ATCC 25922),* Klebsiella pneumoniae* (ATCC 15380),* Pseudomonas aeruginosa* (ATCC 27853), and* Salmonella typhi* and fungi including* Aspergillus clavatus* (KACC 40071),* A. fumigatus* (KACC 40080),* A. niger* (KACC 40280),* A. oryzae* (KACC 44823),* Botrytis cinerea* (KACC 40573),* Candida albicans* (KACC 30003),* C. lunata* (KACC 40392),* Epidermophyton floccosum* (KACC 44918),* Fusarium oxysporum* (KACC 40051),* Gibberella moniliformis* (KACC 44022),* Penicillium chrysogenum* (KACC 40399), and* Trichophyton mentagrophytes* (KACC 45479) were used for the experiment. The clinical strains used in this work are our laboratory collection and the fungal strains were obtained from Korean Culture Collections. The multiple drug resistant (clinical pathogens) strains of* Staphylococcus aureus *were provided by Madras Medical College, Chennai, India.

#### 2.3.2. Cultivation of Bacteria

The bacterial strains were grown in 250-mL Erlenmeyer flasks containing 50 mL MH (Muller Hinton) broth at 37°C on an orbital incubator shaker. The culture flasks were inoculated to 0.1 OD_600_ with freshly prepared cells grown in MH medium under the same culture conditions. The midlog phase cultures were used for the antibacterial study.

#### 2.3.3. Cultivation of Fungi

The filamentous fungi were grown on Sabouraud Dextrose Agar (SDA) slants at 28°C for 7 days. After complete growth the spores were collected using sterile ice cold doubled distilled water and homogenized for the antifungal study.

#### 2.3.4. Minimum Inhibitory Concentration (MIC)

The minimum inhibitory concentration of the compound was performed according to the reference method [10]. The compound was dissolved in water together with 2% dimethyl sulfoxide (DMSO). The initial test concentration (0.5 mg/mL) was serially diluted twofold. Each well was inoculated with 5 *μ*L of suspension containing 10^8^ CFU/mL of bacteria and 10^4^ spore/mL of fungi, respectively. For bacteria, the plates were incubated for 24 h at 37°C, whereas for fungi, the plates were incubated for 24, 48, or 72 h at 30°C. Streptomycin, ketoconazole, and DMSO were used as positive and negative controls, respectively. Five *μ*L of tested broth was placed on the sterile MHA plates and sealed in plastic bags to avoid contamination in the laboratory and at respective temperature. The MIC for bacteria was determined as the lowest concentration of the compound inhibiting the visual growth of the test cultures on the agar plate. For fungi, the MICs were calculated after an incubation time with no visible growth. The experiment was conducted in triplicate.

#### 2.3.5. Fungal Biomass Inhibition Effect

The fungal biomass inhibition effect of the compound was determined by following the method of Valan Arasu et al., 2013 [11]. Briefly, 50 mL of SD broth mixed with 1 mg/mL of the compound was placed in 50-mL flasks and inoculated in triplicate with each test fungus. The fungal strains were incubated at 30°C for 5 days. Flasks without compound were the positive control. After the incubation, fungal growth was measured by harvesting the cells, which were air-dried on preweighed Whatman number 1 filter paper. Average fungal biomass was calculated for each test fungus and compared with the fungal biomass of positive controls.

### 2.4. Antibiofilm Activity

The antibiofilm activity of the compound was determined by following the method of Rejiniemon et al., [10].* E. coli*,* S. typhi*, and* P. aeruginosa *were used as the reference strain.

### 2.5. *In Vitro* Antioxidant Assays

#### 2.5.1. Reducing Ability Assay

The reducing power of the compound was evaluated by modifying the method [12]. Different amounts of the compound (20–100 *μ*g/mL) were suspended in distilled water and mixed with 2.5 mL of 0.2 M phosphate buffer (pH 6.6) and 2.5 mL of 1% K_3_Fe(CN)_6_. The mixture was incubated at 50°C for 20 min; 2.5 mL of 10% TCA was added to the mixture and centrifuged at 3000 rpm for 10 min. The upper layer of the solution (2.5 mL) was mixed with distilled water (2.5 mL) and FeCl_3_ (0.5 mL, 0.1%), and the absorbance was measured at 700 nm. Increase in absorbance of the reaction mixtures indicated increasing of reducing power. Butylated hydroxytoluene (BHT) and vitamin C were used as standards.

#### 2.5.2. DPPH Scavenging Assay

The DPPH scavenging ability of the compound was measured according to the method [13]. Briefly, an ice cold methanol DPPH solution (0.15%) was mixed with serial dilutions (20–100 *μ*g/mL) of the compound under dark environment for 30 min, and the absorbance was read at 515 nm. The antiradical activity was expressed as IC_50_ (*μ*g/mL) (the antiradical dose required to cause a 50% inhibition). Vitamin C was used as standard. The ability to scavenge the DPPH radical was calculated by the following formula:

(1) DPPH  radical  scavenging  activity  %=A0−A1A0∗100,

where *A*_0_ is the absorbance of the control at 30 min and *A*_1_ is the absorbance of the sample at 30 min. All samples were analyzed in triplicate.

#### 2.5.3. Hydroxyl Radical Scavenging Activity

The hydroxyl scavenging assay was performed as described by the method of Sunil et al. [14]. All solutions were prepared freshly. One milliliter of the reaction mixture contained 100 *μ*L of 28 mM 2-deoxy-2-ribose (dissolved in phosphate buffer, pH 7.4), 500 *μ*L solution of various concentrations of the compound (20–100 *μ*g/mL), 200 *μ*L of 200 *μ*M FeCl_3_ and 1.04 mM EDTA (1 : 1 v/v), 100 *μ*L H_2_O_2_ (1 mM), and 100 *μ*L ascorbic acid (1 mM). After an incubation period of 1 h at 37°C, the extent of deoxyribose degradation was measured by the TBA reaction. The absorbance was read at 532 nm against the blank solution. Vitamin C was used as a positive control. The scavenging activity was calculated by formula (1).

#### 2.5.4. Superoxide Radical Scavenging Assay

This activity was measured using NBT (tetrazolium reagent) method as described by Sunil et al. [14]. The method is based on the generation of superoxide radical (O_2_^−^) by autooxidation of hydroxylamine hydrochloride in the presence of NBT, which gets reduced to nitrite. Nitrite in the presence of EDTA gives a color that was measured at 560 nm. Test solutions of the compound (20–100 *μ*g/mL) were taken in a test tube. To this, reaction mixture consisting of 1 mL of (50 mM) sodium carbonate, 0.4 mL of (24 mM) NBT, and 0.2 mL of 0.1 mM EDTA solutions was added to the test tube and immediate reading was taken at 560 nm. About 0.4 mL of (1 mM) of hydroxylamine hydrochloride was added to initiate the reaction; then reaction mixture was incubated at 25°C for 15 min and reduction of NBT was measured at 560 nm. BHT and vitamin C were used as standards. The decreased absorbance of the reaction mixture indicates increased superoxide anion scavenging activity. Absorbance was recorded and the percentage of inhibition was calculated using formula (1).

### 2.6. Anticancer Activity

The anticancer activity of the compound was determined by following the method of Rejiniemon et al. [10].

## 3. Results

**Figure 1:**
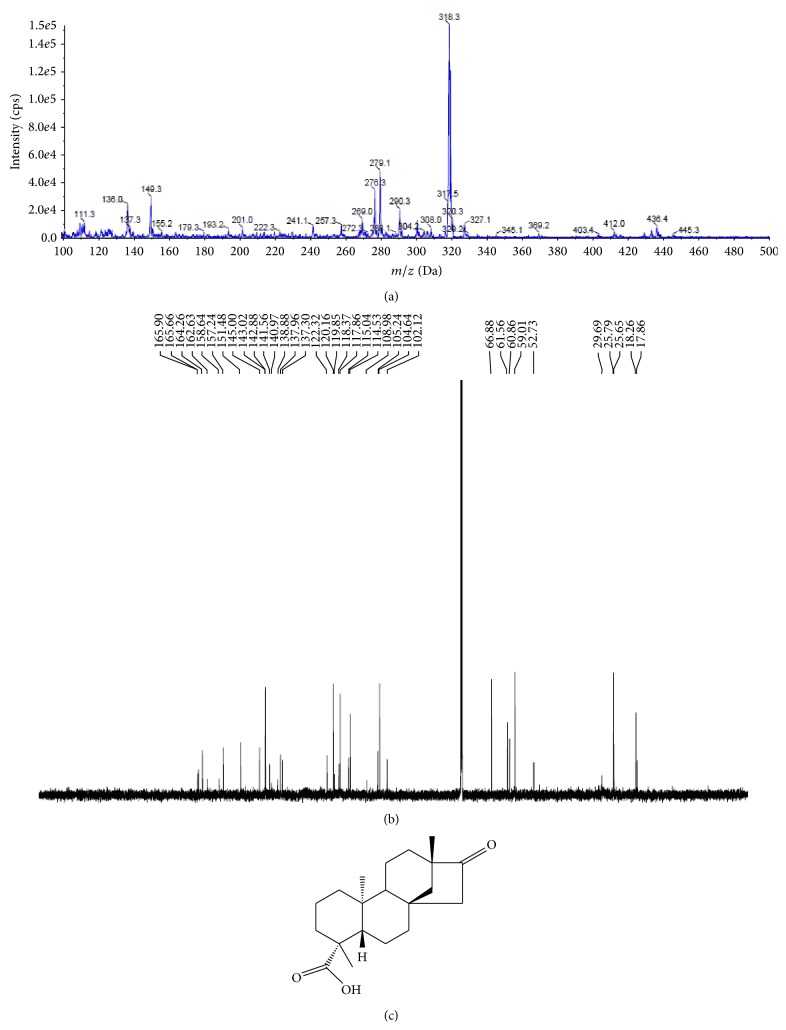
Spectral identification and chemical structure of the identified compound isolated from* Pittosporum tetraspermum*: (a) the mass spectrum of the isolated compound, (b) the NMR spectrum of the isolated compound, and (c) the molecular structure of the isosteviol.

*Pittosporum tetraspermum* is one of the rarely studied medicinal plants found in the unexplored region of Western Ghats forest of Tamil Nadu. The application of this medicinal plant was studied by evaluating its* in vitro* antimicrobial, antibiofilm, anticancer, and antioxidant properties. Primary screening of the different crude organic extracts indicated that the crude ethyl acetate extract showed the significant activity against the Gram positive and Gram negative bacterial pathogens. Disc diffusion method revealed comparatively better activity in ethyl acetate extract than the other extracts (data not shown). Therefore, the crude ethyl acetate extract was purified by column chromatography using different proportions of hexane and ethyl acetate. The collected fraction was tested for its antimicrobial activity and further purified by preparative thin layer chromatography for the identification of the pure compound. The HPLC chromatogram of the identified compounds confirmed the compounds' purity. LC/ESI-MS/MS analysis showed a major fragmentation at 318 (Figure 1(a)). IR spectrum showed a strong absorption band at 996 and 830 regions which revealed the presence of the hydroxyl double bond. ^1^H NMR spectrum exhibited signals at 0.85, 80.89, 0.91, 0.99, 1.2, and 2.3 and the ^13^C NMR spectrum showed varying signals with respect to the carbon region, namely, at 40.1, 80, 121, 140, 135.5, 131, 129, 124, and so forth (Figure 1(b)). These signals confirmed the presence of hydroxyl, methyl group in the structure of the metabolite. These data lead to drawing the molecular formula C_20_H_30_O_3_ and named it isosteviol (Figure 1(c)).

### 3.1. Antibacterial Activity of the Compound

**Figure 2:**
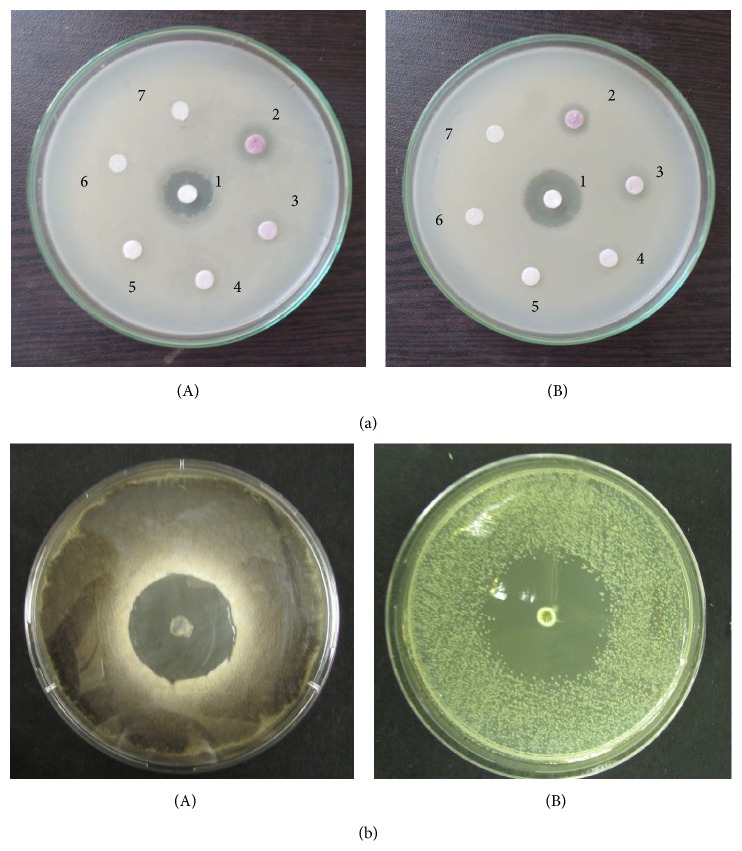
(a) Antibacterial and antifungal activity of isosteviol against microbial pathogens. (a) Antibacterial activity against bacteria. (A)* Klebsiella pneumoniae*; (B)* Staphylococcus aureus*. 1: Streptomycin (positive control); 2: 125 *μ*g/mL; 3: 62.5 *μ*g/mL; 4: 31.25 *μ*g/mL; 5: 15.6 *μ*g/mL; 6: 7.8 *μ*g/mL concentration of the isosteviol; 7: DMSO (negative control). (b) Antifungal activity of isosteviol against fungi. The antifungal activity of the compounds was evaluated by disc diffusion method. (A)* Aspergillus niger*; (B)* Candida albicans*.

The minimum inhibitory concentration (MIC) of the isosteviol against Gram positive and Gram negative pathogens was studied by the broth microdilution method and results are mentioned in Table 1. The results revealed that Gram negative bacteria were more prone to the action of the compound compared to the Gram positive bacteria (Figure 2). Among the Gram positive bacteria,* S. epidermidis* and* S. aureus* showed MIC (125 *μ*g/mL); interestingly the Gram negative bacteria* E. coli *and* P. aeruginosa *shared MIC values of 62.5 *μ*g/mL, respectively, whereas* K. pneumoniae *documented 125 *μ*g/mL. Streptomycin showed better MIC values in comparison to the compound. The MIC values of the multidrug resistant* S. aureus* ranged from 8 to 12 mg/mL.

### 3.2. Antifungal Activity

The MIC values of the compound against filamentous and dermatophytic fungi were displayed in Table 2. Among the filamentous fungi,* P. chrysogenum* exhibited the least MIC values (62.5 *μ*g/mL) and other fungi such as* A. niger*,* A. oryzae*,* C. lunata*,* F. oxysporum*, and* G. moniliformis* growth was completely inhibited at 125 *μ*g/mL concentration (Figure 2(b)).* T. mentagrophytes* showed MIC at 500 *μ*g/mL. Ketoconazole exhibited MIC values ranging within 25–100 *μ*g/mL, respectively.

### 3.3. Fungal Biomass Inhibition Effect

**Figure 3:**
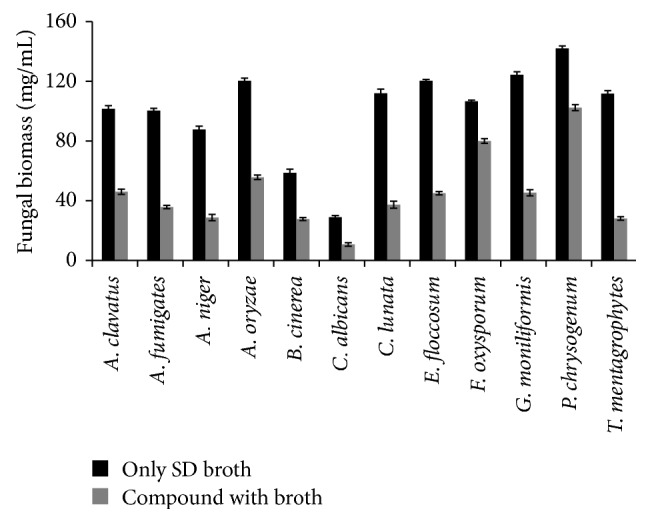
Fungal biomass inhibition effect of isosteviol. The fungal biomass inhibition effect was determined in percentage.

The fungal biomass inhibition effect of the compound was shown in Figure 3. The incubation was 3 or 5 days. After the incubation at 30°C for three days, the compound significantly inhibited the growth of fungi, compared with that of SD controls on dry weight measurements of fungal biomass. The greatest antifungal growth inhibitory activity of the compound was recorded against* F. oxysporum* (75%) and* P. chrysogenum *(72%), followed by* B. cinerea* (47%),* A. oryzae* (46.2%),* A. clavatus* (45.3%),* C. albicans* (37.7%), and* E. floccosum* (37%), respectively. The results confirmed that the compound has significant activity against the spoilage fungus growth.

### 3.4. Antibiofilm Activity

**Figure 4:**
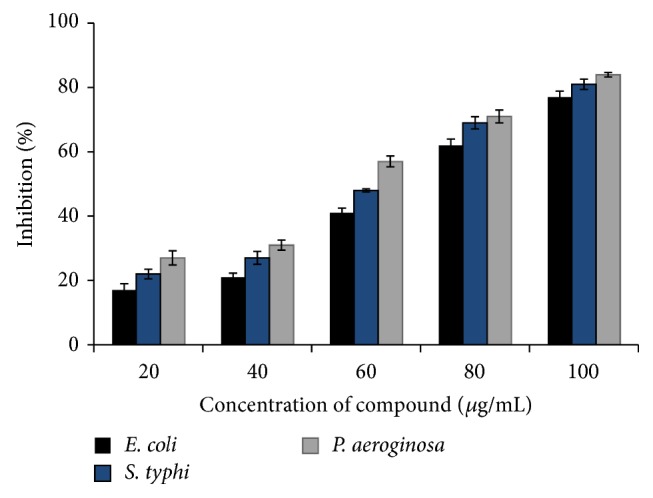
*In vitro* antibiofilm activity of isosteviol. The antibiofilm activity effect was determined in percentage.

*In vitro* antibiofilm activity of the compound against* E. coli*,* S. typhi*, and* P. aeruginosa *was displayed in Figure 4. The antibiofilm activity of the compound was concentration dependent. Results indicated that maximum reductions in cell attachment were observed in* P. aeroginosa* at 100 *μ*g/mL concentration, whereas at 20 *μ*g/mL level the strains exhibited comparatively less biofilm activity.

### 3.5. *In Vitro* Antioxidant Assays

#### 3.5.1. Reducing Ability Assay

**Figure 5:**
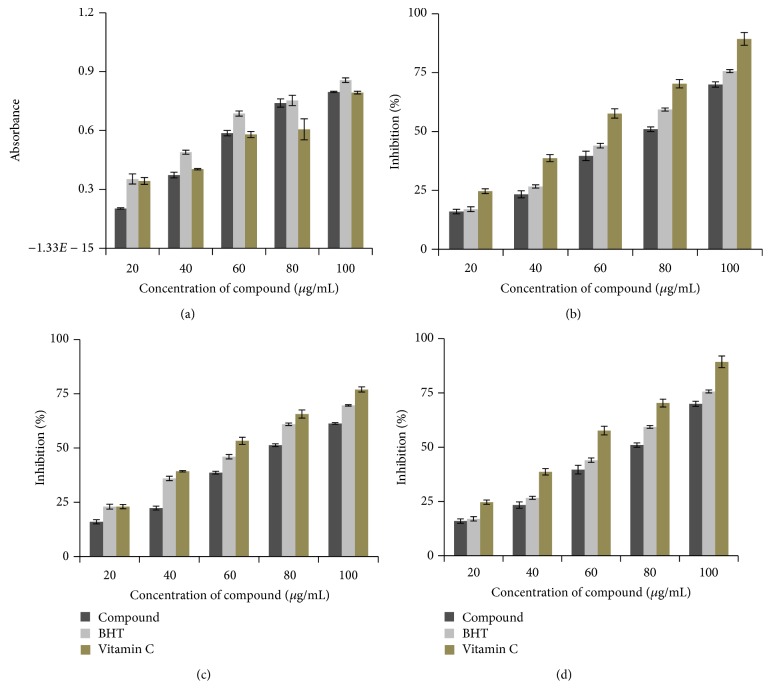
*In vitro* antioxidant activity of isosteviol: (a) reducing ability assay, (b) DPPH scavenging assay, (c) hydroxyl radical scavenging activity, and (d) superoxide radical scavenging assay.

The reducing ability of the compound was compared to the standard BHT and vitamin C (Figure 5(a)). The compound showed comparatively better reducing power and the reducing power increased with increasing concentration.

#### 3.5.2. DPPH Scavenging Assay

The results for DPPH free radicals scavenging assay of the compound are shown in Figure 5(b). Hydroxyl radical scavenging activity exhibited a significant dose dependent manner. The result revealed that the compound had the highest hydroxyl radical scavenging ability, with an inhibition rate of 70% at 100 *μ*g/mL concentration whereas standard BHT and vitamin C exhibited 77% and 90%, respectively, at 100 *μ*g/mL concentration levels.

#### 3.5.3. Hydroxyl Radical Scavenging Activity

The hydroxyl radical scavenging assay results are shown in Figure 5(c). The compound showed the scavenging effect in a concentration dependent manner. The concentrations for 50% inhibition were found to be 76.92, 65.57, and 60 *μ*g/mL for the compound, BHT, and vitamin C, respectively.

#### 3.5.4. Superoxide Radical Scavenging Assay

Superoxide radical scavenging activity of the compound is given in Figure 5(d). The 50% of superoxide anion radical generation was scavenged at the concentration of 91.7 *μ*g/mL. The scavenging activity is directly proportional to the concentration of the compound.

### 3.6. Anticancer Activity

**Figure 6:**
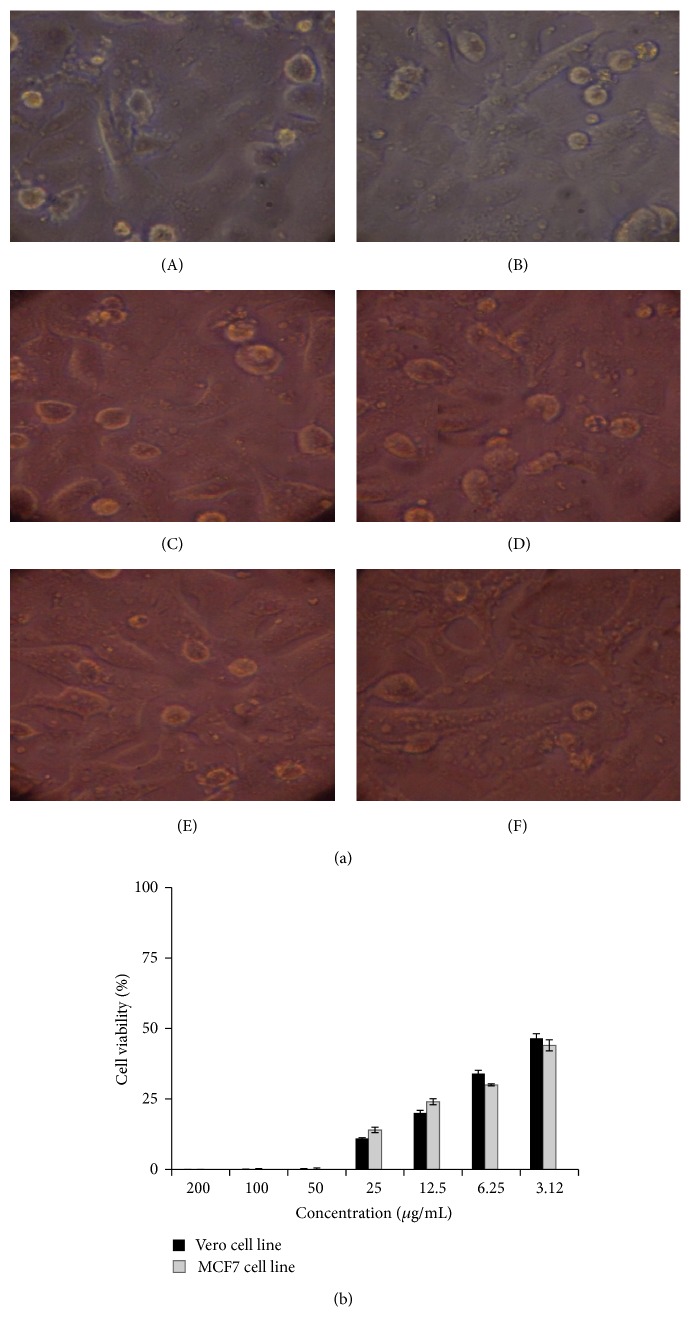
(a) Anticancer activity of isosteviol against Vero cell lines by MTT assay. Cells treated with the compound at different concentrations. (A) 200 *μ*g/mL; (B) 100 *μ*g/mL; (C) 50 *μ*g/mL; (D) 25 *μ*g/mL; (E) 12.5 *μ*g/mL; (F) 6.25 *μ*g/mL. (b) Cytotoxic effect of compound on Vero and MCF7 cell lines as determined by MTT assay.

The anticancer activity of the different concentrations of the compound to a normal Vero cell line and MCF7 cell line was evaluated by MTT assay in 96-well plate. Different concentrations (1.6–200 *μ*g) of the compound on the tested cells exhibited concentration and time dependent inhibition (Figures 6(a) and 6(b)). IC_50_ values were calculated as 2.18 and 2.5 *μ*g for Vero and MCF7 cell lines, respectively. The results indicated that the compound was comparatively nontoxic to normal cells. The cytotoxic activity of compound is at 50 *μ*g levels. This infers that the compound has anticancer activity at lower concentration compared to its cytotoxic activity.

## 4. Discussion

The extensive usage of the commercial antibiotic drugs for the treatment of infectious disease caused by microbial strains without proper medical prescriptions and tests has become a major problem worldwide and also created the environment of the emergence of multiple drug resistance pathogens. Also, modern techniques for development of new antibiotics in the pharmaceutical industry have been pursued by combinatorial chemistry tools which save the time for the synthesis of the molecules but create the environment pollution [15]. Therefore, to overcome this, other strategies include organic synthesis drug pharmacokinetics modification using nanotechnology [16] and search for molecules with unexploited mechanisms of action, often in the form of natural medicines from resources such as plants [17]. It was estimated that at least 12000 active compounds have been isolated from medicinal plants [18, 19]. Forest environment is the biggest reservoir of endangered medicinal plants with chemical and biological diversity. Therefore, research focus on isolation and characterization of novel molecules from the medicinal plants has been gaining importance in recent years because plants are the natural reservoir of many anticancer, antidiarrheal, antibacterial, antifungal, antidiabetic, anti-inflammatory, analgesics, and antifungal agents as well as various therapeutic activities [20]. However, still it has not been fully explored and there is tremendous potential to identify novel molecules with various biological properties.* Pittosporum tetraspermum*, an endangered medicinal plant found in the Western Ghats region, was not studied extensively even though it has some traditional medical applications. In the primary screening the crude extract exhibited significant antimicrobial activity against pathogenic bacteria and spoilage fungi; therefore the crude extracts were column purified and identified the single compound. The IR, LC/MS, ^1^H NMR, and ^13^C NMR of the compound revealed the presence of hydroxyls; double bonds are the characteristics of isosteviol.

From the results of* in vitro* antimicrobial assays against standard strains, it appears that the antibacterial action of the compound is typically more pronounced on Gram negative than Gram positive bacteria. The MIC values ranged within 62.5–125 *μ*g/mL for the tested strains. Among the Gram negative bacteria, the lowest MIC (62.5 *μ*g/mL) was recorded against* P. aeruginosa *and* E. coli *and the highest MIC (125 *μ*g/mL) was recorded against* K. pneumoniae.* It is interesting that the identified metabolites exhibited significant activity against the Gram negative bacteria even though they have an outer lipopolysaccharide membrane that makes the cell wall impermeable to lipophilic solutes. Gram positive bacteria are more susceptible as they have a more permeable outer peptidoglycan layer [21]. The compound also showed comparatively moderate inhibitory activity against the drug resistant* S. aureus.* It showed significant fungal growth inhibition effect against all the tested fungi and the MIC values ranged within 62.5–500 *μ*g/mL. Previously, Roslin and Rosakutty (2012) claimed that the crude butanol extracts of* P. tetraspermum* showed antifungal activity against* F. oxysporum*,* A. niger*, and* Sarocladium oryzae* [22]. The antifungal activity against* A. niger* implied that this plant can be useful for the patients with pulmonary tuberculosis [23].

*Pseudomonas aeroginosa* is associated with biofilm formation on kidney and leads to urinary infections [10, 24, 25]. In the present study the compound revealed significant concentration dependent antibiofilm activity against* P. aeruginosa *which evidenced that the compound is a promising tool for inhibiting the microbial colonization on surfaces and epithelial mucosa which subsequently leads to infections. Reports claimed that number of novel metabolites such as salicylic acid, polyanacardic acid, anacardic acid, polysalicylic acid, catechin, polyphenol, epigallocatechin, and tannic acid from medicinal plants showed antibiofilim activity against* P. aeruginosa* [26, 27].

Antioxidants play an important role by scavenging free radicals and protecting the human body from the external damage caused by free radical induced oxidative stress. Plant metabolites especially those having the phenolic functional group in their chemical structure have been reported to show many useful properties, including anti-inflammatory activity, oestrogenic activity, enzyme inhibition, antiallergic activity, antioxidant activity, vascular activity, and cytotoxic antitumour activity [28, 29]. Inconsistent with the literature, the identified compound exhibited comparatively significant antioxidant activity similar to the synthetic antioxidants, BHT. The chemically synthesized antioxidant is very effective in the medical applications in treating several chronic human diseases such as diabetes mellitus, cancer, atherosclerosis, arthritis, and neurodegenerative diseases as well as aging process but they possess some side effects and toxic properties to human health [30]. Therefore, plant metabolites have attracted many industries. In the present study the identified compound also could be used as chemopreventive and chemotherapeutic agent.

In addition to the above mentioned potentials, the compound exhibited potent inhibitory activity against MCF7 and Vero cell line with an IC_50_ value of 2.5 and 2.18 *μ*g/mL. Results indicated that the compound is nontoxic to cells at very low concentration. Anticancer activities of steviol and isosteviol against human cancer cell lines were already reported [31]. It is understood that the cytotoxic agents may cause necrosis in cells; whereby cells lose membrane integrity leading to cell lysis [32, 33]. Like other identified molecules from the medicinal plants, isosteviol can be used as sources of therapeutically important agents for the treatment of cancer [34, 35].

## 5. Conclusion

Here we have shown that the novel isosteviol identified from endangered medicinal plant* P. tetraspermum* exhibit high inhibitory potency against the bacteria and filamentous fungi. The MIC of the compound ranged from 62.5 to 500 *μ*g/mL for bacteria and fungi. Besides that, it showed significant fungal biomass inhibitory activity against* P. chrysogenum *and* F. oxysporum*. Antibiofilm property of the compound towards pathogens such as* E. coli*,* S. typhi*, and* P. aeruginosa *was its advantage. The anticancer and antioxidant properties would be helpful in preventing or slowing the progress of various oxidative stress-related diseases. However, further hairy root culture studies are necessary to enhance the production of the novel compounds in bulk level.

## Figures and Tables

**Table 1:** Minimum inhibitory concentration of the compound against Gram positive and Gram negative bacteria.

Microorganism	Minimum inhibitory concentration (MIC) (*μ*g/mL)	
	Compound	Streptomycin sulfate
Gram positive		
*Bacillus subtilis* (MTCC 441)	250	2.5
*Staphylococcus aureus* (ATCC 25923)	125	37.5
*Staphylococcus epidermidis * (MTCC 3615)	125	10
*Enterococcus faecalis* (ATCC 29212)	250	25
Gram negative	
*Escherichia coli* (ATCC 25922)	62.5	25
*Klebsiella pneumoniae* (ATCC 15380)	125	25
*Pseudomonas aeruginosa* (ATCC 27853)	62.5	50
Multi drug resistant		
*Staphylococcus aureus* (MMC/3)	>10.00	NT
*Staphylococcus aureus *(MMC/5)	>10.00	NT
*Staphylococcus aureus* (MMC/6)	10.00	NT
*Staphylococcus aureus* (MMC/9)	10.00	NT
*Staphylococcus aureus* (MMC/16)	10.00	NT
*Staphylococcus aureus* (MMC/17)	8.00	NT
*Staphylococcus aureus* (MMC/18)	8.00	NT
*Staphylococcus aureus* (MMC/19)	10.00	NT
*Staphylococcus aureus* (MMC/25)	10.00	NT
*Staphylococcus aureus* (MMC/28)	6.00	NT
*Staphylococcus aureus* (MMC/34)	8.00	NT
*Staphylococcus aureus* (MMC/45)	8.00	NT
*Staphylococcus aureus* (MMC/47)	8.00	NT
*Staphylococcus aureus* (MMC/49)	12.00	NT

MTCC: microbial type culture collection; ATCC: American type culture collection; MMC: strains from Madras Medical College; streptomycin: standard antibacterial agents; compound: isosteviol; NT: not test; for multi-drug resistant strains the concentrations of the compounds were tested in mg/mL level. Streptomycin did not show activity towards multi-drug resistant strains.

**Table 2:** Minimum inhibitory concentration of the compound against fungi.

Microorganism	Minimum inhibitory concentration (MIC) (*µ*g/mL)	
	Compound	Standard
*Aspergillus clavatus *(KACC 40071)	500	50
*Aspergillus fumigates *(KACC 40080)	500	50
*Aspergillus niger *(KACC 40280)	125	25
*Aspergillus oryzae *(KACC 44823)	125	50
*Botrytis cinerea *(KACC 40573)	250	50
*Candida albicans *(KACC 30003)	62.5	100
*Curvalaria lunata *(KACC 40392)	125	50
*Epidermophyton floccosum *(KACC 44918)	250	37.5
*Fusarium oxysporum *(KACC 40051)	125	25
*Gibberella moniliformis *(KACC 44022)	125	100
*Penicillium chrysogenum *(KACC 40399)	62.5	25
*Trichophyton mentagrophytes* (KACC 45479)	500	50

KACC: Korean type culture collection; compound: isosteviol; fungal control reference (ketoconazole: *μ*g/mL).
